# Self-motion facilitates echo-acoustic orientation in humans

**DOI:** 10.1098/rsos.140185

**Published:** 2014-11-12

**Authors:** Ludwig Wallmeier, Lutz Wiegrebe

**Affiliations:** 1 Division of Neurobiology, Department Biologie II, Ludwig-Maximilians-Universität München, Großhadernerstr. 2, 82152 Planegg-Martinsried, Germany; 2 Graduate School of Systemic Neurosciences, Ludwig-Maximilians-Universität München, Großhadernerstr. 2, 82152 Planegg-Martinsried, Germany

**Keywords:** auditory, echolocation, binaural hearing, temporal processing

## Abstract

The ability of blind humans to navigate complex environments through echolocation has received rapidly increasing scientific interest. However, technical limitations have precluded a formal quantification of the interplay between echolocation and self-motion. Here, we use a novel virtual echo-acoustic space technique to formally quantify the influence of self-motion on echo-acoustic orientation. We show that both the vestibular and proprioceptive components of self-motion contribute significantly to successful echo-acoustic orientation in humans: specifically, our results show that vestibular input induced by whole-body self-motion resolves orientation-dependent biases in echo-acoustic cues. Fast head motions, relative to the body, provide additional proprioceptive cues which allow subjects to effectively assess echo-acoustic space referenced against the body orientation. These psychophysical findings clearly demonstrate that human echolocation is well suited to drive precise locomotor adjustments. Our data shed new light on the sensory–motor interactions, and on possible optimization strategies underlying echolocation in humans.

## 2. Introduction

Some animals, like bats and toothed whales, are known to use echolocation for orientation and navigation purposes. They actively emit precisely timed acoustic signals and analyse the resulting echoes to extract spatial information about their environments. This allows them to compensate for a lack of visual stimuli due to nocturnal darkness or murky waters in their habitats [1,2]. Some blind humans also use echoes from self-generated sounds to represent their spatial environment with high precision (for reviews, see [3,4]). It has been shown that using echolocation humans can detect obstacles [5–7], discriminate between objects of different texture or size [8], localize a sound-reflecting surface [9] and estimate its distance [8,10]. There is accumulating evidence that even naive sighted subjects can be easily trained to reach high acuity in certain echolocation tasks [11,12]. However, little is currently known about the sensory–motor interactions underlying effective echo-acoustic orientation and navigation behaviour.

In daily life, humans are confronted with diverse auditory information that they have to structure into meaningful streams to guide behaviour, which is called ‘auditory scene analysis’ [13]. This perceptual organization of streams is not instantaneous, but builds up with experience over time and can break down when the auditory scene changes suddenly [14]. Kondo *et al.* [15] investigated the effects of self-motion on auditory scene analysis, and found that the perceptual organization breaks down immediately after movement onset, but is rapidly and more accurately reorganized afterwards due to the time-varying binaural cues that motion provides. This indicates that sensory–motor interactions influence auditory scene analysis, which raises the expectation that echo-acoustic orientation could also be a dynamic process that benefits from self-motion.

Empirical findings on the influence of motion on echolocation in humans are scarce and sometimes inconsistent. Rosenblum *et al.* [16] reported a subtle advantage of linear approaching motion for their human subjects in an echo-acoustic distance estimation experiment with self-generated vocalizations. However, Ashmead & Wall [17] found no significant effect of listener movement on echo-acoustic object detection when they simulated linear approaching motion in virtual space with pre-recorded stimuli. These results suggest that the vestibular and/or proprioceptive component of self-motion—which was absent in the latter study—could be required to benefit from movement during echolocation.

Other types of self-motion, like head rotations, may have a stronger effect on echolocation performance than linear approaching motion. Porpoises are known to use large head movements to accentuate binaural differences in the perceived echoes and thereby enhance echolocation accuracy [18–20]. Similar behaviour has been found in Egyptian fruit bats, which do not centre their sonar beam directly on the target when localizing but oscillate it laterally around the target [21]. There are hints that blind humans also profit from head rotations during echolocation: Kellog [8] and Rice [22] reported that blind subjects spontaneously executed periodic yaw movements of their heads by up to ±45^°^ in echolocation experiments. However, a formal quantification of the interactions between rotational self-motion and echo-acoustic sensitivity in humans is not available today.

Several studies showed improved performance in sound source localization tasks due to active or passive head rotations, especially with respect to horizontal accuracy and front–back disambiguation [23–28]. However, it is not *a priori* clear whether these findings also apply in an echolocation context. Thaler *et al.* [29,30] found differing activation patterns in temporal–occipital cortex when subjects listened either to a moving sound source or to a moving sound reflector. This suggests a functional segregation of processing of auditory source-motion and echo-motion. Therefore, research is needed to characterize the influence of motion on echolocation performance.

This study investigates the influence of rotational self-motion on echo-acoustic orientation in humans. All experiments were conducted in virtual echo-acoustic space (VEAS) using the binaural room impulse responses (BRIRs) of a real corridor: to explore the virtual environment, subjects produced echolocation calls which were picked up by a headset microphone, convolved in real time with the respective BRIR, and played back via headphones.

## 3. Material and methods

### 3.1 Subjects

Eight sighted subjects participated in the study (23.5±2.2 years of age (mean ± s.d.), one female). All subjects had hearing thresholds of less than 10 dB hearing level for both ears for all tested frequencies (250–8000 Hz in octave steps). To provide a proof of concept for our VEAS presentation and to compare our sighted subjects’ performance with that of blind echolocation experts, we also tested two blind professional echolocation teachers. Both were blind since at least infancy, taught themselves to echolocate during childhood, and since then have been using echolocation on a daily basis.

### 3.2 Stimuli and apparatus

**Figure 1.**
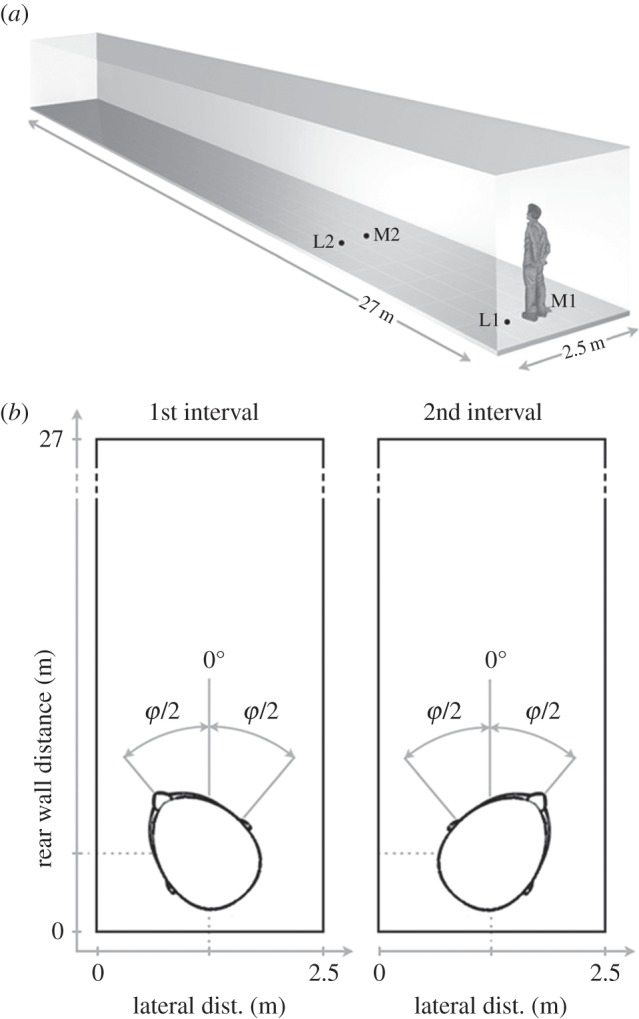
(*a*) Illustration of the virtual corridor. Echo-acoustic orientation performance was tested at two positions on the midline of the corridor (positions M1 and M2 at rear wall distances of 75 and 700 cm, respectively) and two positions 75 cm from the left lateral wall (positions L1 and L2 at rear wall distances of 75 and 700 cm, respectively). (*b*) Illustration of Experiment 1. In a 2AIFC paradigm, subjects were asked to discriminate between a leftward and a rightward deviation from the virtual corridor’s longitudinal axis (0^°^).

Subjects gathered spatial information about their environment for orientation by listening to echoes of their own vocalizations. All experiments were conducted in VEAS using the BRIRs of a real corridor with a constant width of 2.5 m, a length of 27 m and a height of 4 m (cf. figure 1*a*). The side walls and the ceiling of the corridor were made of concrete and the flooring consisted of PVC. The recording of the BRIRs is described later.

During the experiments, subjects were seated in a sound-attenuated anechoic chamber with a size of 2.0×2.0×2.2 m (Industrial Acoustics Company GmbH, Niederkrüchten, Germany). The walls of the chamber were lined with 20-cm acoustic wedges, which decreased the level of echoes by at least 40 dB for frequencies higher than 500 Hz. To present a specific position and orientation in VEAS, the subjects’ vocalizations were recorded anechoically with a headset microphone (Sennheiser HS2-EW, Wedemark, Germany), convolved in real time with the respective BRIR, and then presented via headphones (K701, AKG Acoustics GmbH, Vienna, Austria). Parts of the headphones’ ear cups were removed, which allowed for undisturbed perception of the direct sound from the mouth to the ears via the free field and of the echoes via headphones at the same time.

The BRIRs presented to the subjects had a length of 2.7 s and were all derived from the BRIRs recorded in the real corridor, while compensating for the frequency response characteristics of the microphone and the modified headphones. The headphones and the microphone were connected to a personal computer (PC with Windows 7) with an external soundcard (MOTU Audio 24I/O, Cambridge, MA, USA) via wireless transmitter and receiver systems (Sennheiser EW 172 G3 and EW 300 IEM G3, Wedemark, Germany). On the PC, a real-time convolution kernel (Soundmexpro, Oldenburg, Germany) was run under Matlab. The overall delay of the convolution engine was 3.3 ms. In order to guarantee the correct time delays for the echoes, the first 3.3 ms of the BRIRs were cut. Since the direct sound from the mouth to the ears was not simulated and all virtual reflecting surfaces were at a distance of at least 75 cm from the subject (corresponding to an echo delay of at least 4.4 ms), no important information was lost when cutting the first 3.3 ms of the BRIRs.

The authentic reproduction of the corridor’s acoustics was verified by measuring the BRIRs of the VEAS using the same recording set-up and procedure as for the original BRIR acquisition. Additionally, two blind echolocation experts validated the VEAS presentation perceptually: they took part in the experiments in VEAS and afterwards they went to the original real corridor to compare the acoustic impressions. Both blind experts successfully solved the echo-acoustic tasks in VEAS without any specific training and they confirmed that the acoustic impression in VEAS and in the real corridor were highly consistent.

### 3.3 Acoustic recordings

For the BRIR recordings, a head-and-torso simulator (HATS, B&K 4128C, Brüel & Kjær Instruments, Nærum, Denmark) was used as the core part of a custom built mobile recording set-up. The HATS was attached to a computer-controlled turntable which was mounted on a small wooden cupboard with wheels. The recording was controlled via a notebook computer connected to an external soundcard (ProFire 610, M-Audio, Willich, Germany).

The corridor was acoustically excited with a 10-s logarithmic sine sweep with a frequency range from 200 to 20 000 Hz. The sweep was created with Matlab (The MathWorks, Inc., Natick, MA, USA), amplified (Amplifier A-109, Pioneer Electronics, Willich, Germany) and transmitted to the loudspeaker in the mouth of the HATS. The emitted signal and its reflections were then recorded via the microphones in the ear-canals of the HATS and amplified with a Brüel & Kjær Nexus conditioning amplifier. Playback and recording were implemented with SoundMexPro (HörTech GmbH, Oldenburg, Germany). The loudspeaker in the mouth of the HATS was calibrated by filtering the playback signal with a respective compensation impulse response. This guaranteed that the frequency response of the loudspeaker had a flat spectrum, while the sound emission characteristics of the HATS (as a function of frequency, azimuth and elevation) were preserved. With an overall height of 180 cm, the recording set-up was appropriate to simulate a human adult in upright position who actively vocalized with his mouth and perceived his own vocalizations both directly from the mouth to the ears (direct sound) and from the mouth via reflections to the ears (echo).

To extract the BRIRs, the emission and the binaural recording were cross-correlated and afterwards filtered to compensate for the logarithmic sweep. This procedure was used to acquire the BRIRs for two positions on the midline of the corridor (positions M1 and M2 at rear wall distances of 75 and 700 cm, respectively; cf. figure 1*a*) and two positions in close proximity of 75 cm to the left lateral wall (positions L1 and L2 at rear wall distances of 75 and 700 cm, respectively). At each position, measurements were conducted for 90 orientations with an angular resolution of 4^°^. Finally, the BRIRs were interpolated (by linear interpolation of the linear magnitude spectra and unwrapped phase spectra) to increase the angular resolution from 4^°^ to 0.2^°^.

### 3.4 Procedure for Experiment 1

In a two-alternative, two-interval, forced-choice (2AIFC) paradigm, subjects were trained to discriminate echo-acoustically between two orientations in VEAS, which were symmetrically centred around the virtual corridor’s longitudinal axis (cf. figure 1*b*). Each trial started with a 50-ms, 1-kHz tone pip to indicate the beginning of a 2-s exploration interval. During the exploration interval, a BRIR was presented as described above, i.e. the subjects vocalized and listened to the computer-generated echoes of their own vocalizations. The ending of the exploration interval was signalled by a 2-kHz tone pip. After a 500-ms pause, the second exploration interval was presented in the same way, but with a different BRIR. Subsequently, the subjects had to indicate (using a joystick) whether the first or the second exploration interval contained the orientation towards the right-hand side of the corridor’s longitudinal axis. Subjects were given audio feedback by a 250-ms frequency chirp which was upward modulated for positive feedback and downward modulated for negative feedback.

The azimuthal separation of the two presented orientations (illustrated in figure 1*b* as the angle *φ*) was adapted with a three-down–one-up procedure: it was decreased after three correct responses and increased after one incorrect response, which yields threshold estimates at the 79.4% correct level [31]. Until the third reversal of the adaptive track, *φ* was changed by a factor of 2. It was changed by a factor of 1.2 for reversals four and five, and by 1.1 from the sixth reversal on. The experimental run was stopped at the 11th reversal and the just noticeable difference (JND) was calculated as the geometric mean of the azimuthal separations in degrees at the last six reversals of the run. All subjects were trained until their performance stabilized over runs. The criterion for stable performance was fulfilled when the standard deviation across the last three runs was less than 25% of the mean across these runs.

### 3.5 Procedure for Experiment 2

In Experiment 2, subjects were asked to align themselves to be as parallel as possible to the virtual corridor’s longitudinal axis (which is the 0^°^ direction in figure 1*b*), starting from a random orientation. Depending on the specific condition, rotations were conducted via rotation of the virtual corridor relative to the subject’s fixed body and head (Experiment 2.1), via rotation of the subject’s whole body in a rotating chair relative to the fixed virtual corridor (Experiment 2.2) or via independent rotation of both the subject’s head and body relative to the fixed virtual corridor (Experiment 2.3). Data acquisition was randomly interleaved to balance residual training effects.

In Experiment 2.1, subjects used a joystick connected to the PC to vary the angular velocity as a function of the joystick’s deflection. The top speed was limited to 30^°^ s^−1^ for rotation. In Experiment 2.2, subjects used the joystick to control the relative motion of themselves and the VEAS in the same way as in Experiment 2.1, but the VEAS was fixed in world coordinates and the chair the subject was sitting on was rotated via a computer-controlled driving mechanism. The current orientation was assessed via a tracking system (Intersense motion tracker, Billerica, MA, USA) that acquired the chair’s orientation 10 times per second. The BRIRs were updated with the same frequency of 10 Hz according to the tracked orientation. In Experiment 2.3, free head movements were allowed in addition. The current orientation was assessed by tracking the orientation of the subject’s head 10 times per second. The orientation of the rotating chair was also tracked in order to document the relative orientation of head and body.

Before each experimental run, the sighted subjects were blindfolded and the light was turned off. Subjects then started the experiment by pressing a button on the joystick. Subsequently, a short tone pip (50 ms, 1 kHz) indicated that VEAS presentation was switched on. Subjects could explore VEAS via echolocation and rotation without time limit. When they felt confident that they had found the required orientation, they pressed another button on the joystick, which stopped the experimental run. Another tone pip (50 ms, 2 kHz) confirmed that the run was stopped.

In each experimental run, the subjects’ orientation in VEAS was written to a file and saved to hard disk once per second. All subjects were trained in each condition until their performance stabilized over runs. The stability criterion was that the standard deviation across the last three runs was smaller than 5^°^. The overall performance of a subject was defined as the mean value of the performance in the last three runs in degrees.

### 3.6 Control experiment

A control experiment was conducted in order to investigate the influence of rotation speed, which was limited in Experiments 2.1 and 2.2, but not in Experiment 2.3. The control experiment was identical to Experiment 2.1, except that the speed limit for rotation of the VEAS was increased to 360^°^ s^−1^, which is faster than the highest speed observed in Experiment 2.3.

## 4. Results

**Figure 2.**
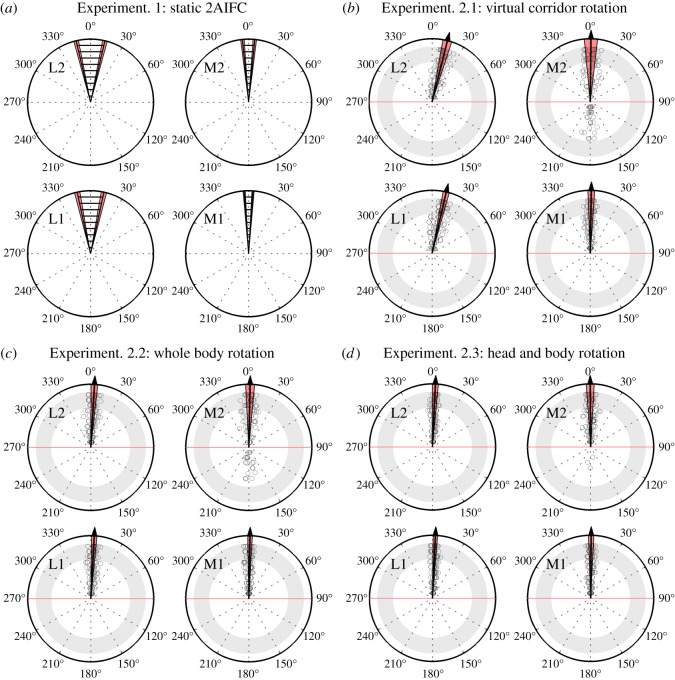
Results from Experiments 1 and 2 in terms of deviation from the required orientation (0^°^). The rows and columns represent the two rear wall distances and the two lateral wall distances, respectively. (*a*) In Experiment 1, the average JND (striped area, standard deviation red) was significantly smaller in the middle of the corridor than close to a lateral wall. (*b*–*d*) Final orientation for all subjects in the last 10 runs of Experiment 2 (increasing radius encodes the chronological order). Orientation performance was calculated for each subject by averaging over the last three runs (in the grey area). The mean and standard deviation across subjects is represented by the arrows and the red areas, respectively. (*b*) Experiment 2.1 confirmed the detrimental effect of a nearby lateral wall and showed that it was due to a systematic bias. (*c*) This systematic error was absent in Experiment 2.2, indicating that subjects benefited from the whole-body rotation. However, they still made systematic front–back confusion errors at position M2 (orientations below the red line). (*d*) These could well be resolved in Experiment 2.3, when additional head rotations were allowed.

**Figure 3.**
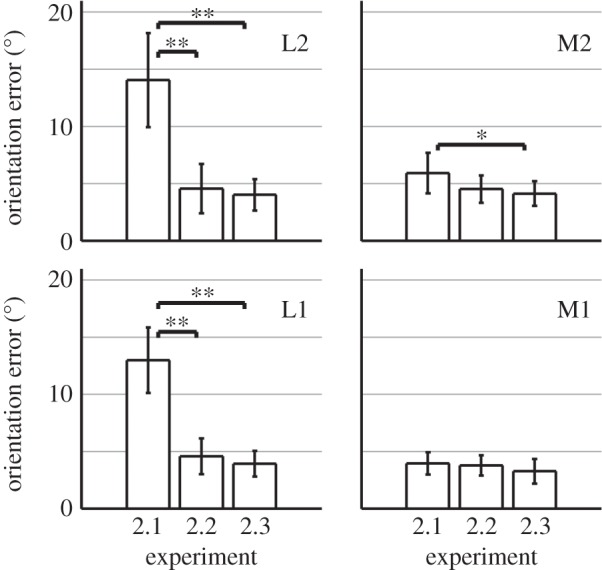
Orientation errors from Experiment 2 in terms of absolute deviation from the required orientation (0^°^). Bars and error bars represent averages and standard deviations across all sighted subjects, respectively. The asterisks highlight significant differences between pairs of experimental conditions (Wilcoxon rank-sum test, *p<0.05, ^**^p<0.01). When a lateral wall was nearby (positions L1, L2), performance was significantly better in Experiments 2.2 and 2.3, where self-motion was allowed, than in Experiment 2.1, where subjects were stationary. This shows that subjects profited from self-motion in the echo-acoustic orientation task.

All subjects were successfully trained to perform the echo-acoustic orientation tasks in VEAS. The analysed results are shown in figures 2 and 3. In addition, all raw data that is necessary to conduct the analyses and draw the figures presented in the paper is provided in the electronic supplementary material. In order to ensonify their virtual echo-acoustic environment, all subjects produced short broadband tongue clicks. Typically, the clicks had a duration of 4–11 ms and a sound pressure level of 60–90 dB SPL as measured at the headset microphone. The peak frequencies of the clicks ranged from 3 to 9 kHz. In the 2AIFC experiment, subjects typically produced two to seven clicks per 2-s interval.

### 4.1 Experiment 1: static 2AIFC

Experiment 1 aimed to formally quantify the ability of humans to use the echoes of self-generated vocalizations for orientation. In a 2AIFC paradigm, eight naive sighted subjects were successfully trained to discriminate a leftward deviation from a rightward deviation from the required orientation (cf. figure 1*b*). Here, subjects’ orientation and position in real space were always fixed. Subjects performed best when positioned on the virtual corridor’s midline (cf. figure 2*a*): for positions M1 and M2, the average JND was 8.1^°^ and 11.3^°^, respectively. However, performance deteriorated significantly when the left lateral wall was closer than the right one: for positions L1 and L2, the average JNDs were 24.5^°^ and 26.4^°^, respectively. A two-way ANOVA revealed a significant effect of lateral wall distance (*F*_1,28_=138.88, *p*<0.001), but no significant effect of rear wall distance (*F*_1,28_=3.60, *p*=0.07).

The two blind echolocation experts both managed to solve the task without any training, which is a proof of concept for our VEAS implementation. Their average JNDs were 7.4^°^ in the middle of the corridor and 16.1^°^ near a lateral wall. For all positions, the performance of the experts was slightly better than that of the sighted subjects. However, the trends in terms of a significant effect of lateral wall distance and no significant effect of rear wall distance were the same. These results show that naive sighted subjects can be effectively trained to use echolocation for orientation.

### 4.2 Experiment 2: free rotation

Performance for Experiment 2 was quantified in terms of the ‘orientation bias’, defined as the mean deviation from the required orientation (shown in figure 2), and in terms of the ‘orientation error’, defined as the mean absolute deviation (shown in figure 3).

In Experiment 2.1, orientation adjustment was realized by rotating the VEAS relative to the fixed subject. The average orientation error was 4.9^°^ for symmetric lateral wall distances and 13.5^°^ when one lateral wall was nearby (cf. figure 3). There was a significant effect of lateral wall distance (*F*_1,28_=81.87, *p*<0.001), but no significant effect of rear wall distance (*F*_1,28_=2.69, *p*=0.11). These data confirm the detrimental effect imposed by a nearby lateral wall, as observed in Experiment 1. Moreover, the dynamic orientation paradigm revealed that this detrimental effect was due to a systematic error: for all subjects, there was a systematic orientation bias away from the nearby wall, with an average value of 13.5^°^ (cf. figure 2*b*). Although the performance measures used in Experiment 1 (thresholds via 2AIFC with feedback) and Experiment 2.1 (echolocation without time limit and without feedback) are not directly comparable, results from both experiments consistently show that subjects performed better in the middle of the corridor than near a lateral wall. This indicates that in Experiment 2.1, the additional dynamic cues due to VEAS rotation did not help subjects to overcome the detrimental effect imposed by a nearby lateral wall.

In Experiment 2.2, the subject’s whole body was rotated via a computer-controlled rotating chair, whereas the virtual corridor was fixed in world coordinates. Head rotations were not allowed. Here, subjects could exploit vestibular cues for orientation in addition to the dynamic echo-acoustic cues. For symmetric lateral wall distances, the average orientation error was 4.1^°^, which is not significantly different from that for Experiment 2.1 (Wilcoxon rank-sum test, *W*_16,16_=292, *p*=0.30; cf. figure 3). However, here the detrimental effect of a nearby lateral wall was much less pronounced than in the previous experiments: for asymmetric lateral wall distances, the average orientation error was 4.5^°^, which is significantly lower than for Experiment 2.1 (*W*_16,16_=392, *p*<0.001; cf. figure 3). There was no significant main effect of lateral or rear wall distance with respect to orientation bias or orientation error (cf. figure 2*c*). Hence, subjects must have exploited the additional vestibular cues to overcome the systematic bias. This shows that self-motion did indeed facilitate echo-acoustic orientation.

Experiment 2.3 was identical to Experiment 2.2, except that free head rotations were allowed. Therefore, here subjects could exploit proprioceptive cues in addition to echo-acoustic and vestibular cues, and rotation speed was not limited by an intermediate technical device. In terms of orientation bias and orientation errors, performance for Experiments 2.2 and 2.3 did not differ significantly (cf. figures 2 and 3). However, figure 2 shows that, over the last 10 runs, the number of front–back confusions at position M2 was significantly lower in Experiment 2.3 than in Experiment 2.1 (*χ*^2^=20.95, *p*<0.001) and Experiment 2.2 (*χ*^2^=12.33, *p*<0.001).

Analysis of the subjects’ movements in VEAS revealed that subjects changed their strategy in Experiment 2.3. In Experiments 2.1 and 2.2, rotation speed was relatively slow but constant, with periodic interruptions during which subjects produced echolocation calls. Between two adjacent echolocation calls, subjects hardly ever covered a range of more than 5^°^–10^°^. This indicates that subjects tried to orient by continuously scanning the acoustic properties of the virtual corridor. Constant echo-acoustic feedback was needed as a reference to assess VEAS.

In Experiment 2.3, however, subjects moved their head back and forth between certain orientations several times quite fast. This indicates that they tried to compare remote orientations directly, either to use them as landmarks or to disambiguate orientations with similar acoustic properties, like the 0^°^ and the 180^°^ orientation at position M2. This behaviour seems to have helped subjects to avoid front–back confusions in Experiment 2.3. During head rotations, subjects covered angles of up to 180^°^ without interruption by intermediate echolocation calls while they kept the orientation of the rotating chair fixed. This indicates that the additional proprioceptive cues due to head motion allowed subjects to effectively assess echo-acoustic space referenced against the body orientation.

As for Experiment 1, both blind echolocation experts solved the tasks in Experiment 2 without any training, which again confirms the validity of our VEAS implementation. Both experts fulfilled the stability criterion after only three to four runs. The orientation errors were as low as 2^°^ for all conditions, except positions L1 and L2 in Experiment 2.1. Here, orientation was biased by about 8^°^ away from the nearby lateral wall.

### 4.3 Results from the control experiment

A control experiment to Experiment 2.1 was conducted in order to investigate the influence of rotation speed. The results showed that subjects did not use the full range of speeds available in the control experiment, but employed roughly the same average and top speed as in Experiment 2.1. There were no significant differences in performance between the Experiment 2.1 and the control experiment. Hence, high rotation speed alone does not facilitate echo-acoustic orientation. This indicates that the vestibular and proprioceptive components of self-motion are crucial for calibrating the perception of the angle that is covered during rotation.

## 5. Discussion

The experiments show that sighted subjects can be successfully trained to use echo-acoustic cues for orientation in VEAS. The authenticity of our VEAS implementation was verified both physically and psychophysically, namely by measuring and comparing impulse responses for real and for virtual space, and by tests using two blind echolocation experts.

In Experiment 1, subjects had to discriminate between two discrete acoustic snapshots of a virtual corridor. The method of VEAS presentation in combination with a strict 2AIFC paradigm guaranteed that subjects could not exploit any cues other than the intended echo-acoustic ones. They performed quite well as long as the lateral walls were symmetrically arranged. Such a configuration facilitates the exploitation of basic binaural-difference cues, in that subject simply had to judge whether the overall echo was stronger in their left or right ear. The obtained thresholds are consistent with previous studies measuring echolocation acuity in the horizontal plane [9,11]. However, performance deteriorated significantly in close proximity of a lateral wall, although it has been shown that monaural echo-acoustic information due to sound reflections from a wall is most useful at a distance of approximately 1 m or less [32]. This indicates that for the current task, the basic binaural-difference cues were more helpful than monaural absolute-loudness cues.

In Experiment 2, subjects could voluntarily control their orientation in the virtual corridor via rotation of the corridor around their body and head (Experiment 2.1), via rotation of their whole body in a rotating chair relative to the virtual corridor (Experiment 2.2) and via independent rotation of both their head and body relative to the virtual corridor (Experiment 2.3). Results from Experiment 2.1 confirmed the detrimental effect of a nearby lateral wall and showed that it was due to a systematic bias, which is consistent with the results of Dufour *et al.* [33], who reported a biasing effect of nearby walls on sound localization. This indicates that the prominent early reflections from the closer lateral wall may have partially masked later reflections from the opposite lateral wall and thus systematically biased the subjects’ estimate of their orientation in the virtual corridor. This systematic error was absent in Experiment 2.2, indicating that subjects had benefited from the whole-body rotation. However, they still made systematic front–back confusion errors. These were reduced in Experiment 2.3, when additional head rotations were allowed.

The detrimental effect of a nearby lateral wall that we observed in Experiments 1 and 2.1 seems to contradict the results of Shinn-Cunningham & Ram [34]. They investigated how well human listeners can exploit changes in reverberation to identify their location and orientation in a room, and found that listeners reliably hear and exploit monaural spectral cues from prominent, early echoes. However, listeners were relatively insensitive to the exact timing and arrival direction of echoes, especially regarding the pattern of late-arriving echoes. In a second experiment, they showed that binaural echo-acoustic cues even impeded the perception of monaural intensity cues.

Yovel *et al.* [21] described a similar conflict between absolute echo intensity and time-varying echo cues in echolocating bats. They trained Egyptian fruit bats to find a spherical target, to fly towards it and to land on it. During their experiments, Yovel *et al.* measured the directional aim of the bats’ sonar clicks. The authors observed two different phases of spatial localization behaviour: for target detection, the bats first maximized the echo intensity by orienting the peak of their echolocation call directly on the target. For fine-tuning the localization, they then pointed the peak of their calls to the left and to the right of the target in an oscillatory manner. In this way, not the peak but the slope of the call was pointed at the target. Therefore, the intensity of the echoes was not maximal (because the peak of the call was not pointed directly at the target), but time-varying echo cues were emphasized. The authors proposed a trade-off between maximal echo intensity for detection and time-varying cues for localization.

This trade-off may also explain the differences between the current results and those of Shinn-Cunningham & Ram [34]. In the latter study, subjects had to discriminate between four different positions in a room, which differed in the presence or the absence of nearby, sound-reflecting walls. Thus, they used a detection task, for which maximal echo intensity is known to be optimal. In our study, subjects had to fine-tune their orientation relative to the surrounding, sound-reflecting walls. This was a localization task, for which time-varying cues may be more helpful.

Both in the static 2AIFC Experiment 1 and in the dynamic Experiment 2.1 with VEAS rotation, subjects performed better in the middle of the corridor than near a lateral wall, i.e. the detrimental effect of a nearby lateral wall was observed in both experiments. With respect to this detrimental effect, subjects did not profit from the dynamic cues due to rotation of the VEAS around themselves. The results suggest that subjects did not adapt their strategy to exploit dynamic cues, but still compared acoustic snapshots to find the target orientation, just like in the experiment with static cues. This is consistent with the results of Ashmead & Wall [17], who found no significant effect of listener movement on echo-acoustic object detection when they simulated linear approaching motion in virtual space with pre-recorded stimuli, i.e. without vestibular stimulation. In Experiments 2.2 and 2.3, additional vestibular cues induced by self-motion were available to the subjects. Here, performance was significantly better than in the experiments where subjects were stationary. Hence, self-motion facilitates echo-acoustic orientation, and the vestibular component of self-motion is essential for benefiting from movement during echolocation. These results are consistent with the findings of Rosenblum *et al.* [16], who reported a subtle advantage of subject motion in an echo-acoustic target-ranging experiment with vestibular cues available.

The findings of Kondo *et al.* [15] may shed light on the nature of the observed enhancement due to self-motion. They demonstrated that self-motion facilitates the perceptual organization of auditory streams by providing time-varying binaural cues. Our subjects may have profited from the same effect in Experiments 2.2 and 2.3, where self-motion was allowed. It is possible that self-motion helped them to perceptually segregate the reflections of the two lateral walls into individual streams as predicted by Kondo *et al.* [15]. This might have helped them to overcome the bias imposed by a nearby lateral wall.

In Experiments 2.1 and 2.2, subjects made systematic front–back errors, which were reduced in Experiment 2.3, when head motion was allowed. The occurrence of such confusions and the influence of head motion are well documented for sound source localization with non-individualized head-related transfer functions (HRTFs) [35]. However, in the current experiments, front–back confusion only occurred at position M2. This shows that subjects were not confused by the non-individualized HRTFs, but by the acoustic similarity of opposite orientations at this specific position.

When head motion was allowed, subjects moved back and forth between certain orientations several times quite fast, i.e. they overcame the problem of acoustic similarity by comparing remote orientations directly, using a high angular speed of head motion. The psychophysical results confirm that this strategy was more effective than the slow scanning strategy subjects employed in all other conditions. However, subjects went back to slow scanning in the control experiment (where we increased the speed limit of the virtual corridor rotation). So, here, subjects did not exploit the increased speed limit of the rotating chair. This shows that the vestibular and proprioceptive components of self-motion were crucial to calibrate the perception of the angle that was covered during rotation and to create a cognitive map of the virtual corridor.

Kolarik *et al.* [36] have shown that blindfolded sighted subjects are able to use echoic spatial information from a sensory substitution device (SSD) in combination with body-scaled information for accurate motor adjustments of their shoulder position when passing through an aperture. However, the authors point out that human echolocation with self-generated sounds critically differs from using spatial information obtained with an SSD, since SSDs produce ultrasound and have built-in signal processing, whereas human echolocation involves comparing sound emission and echo. They conclude that it remains to be determined whether echolocation with self-generated sounds can be used for tailoring precise motor adjustments. A recent review of the literature on human echolocation [37] affirmed the need for further work with respect to locomotive guidance through echolocation. The current results show that human subjects can use echo-acoustic information from self-produced vocalizations to turn their body and/or head to a desired heading with high accuracy. This shows that active human echolocation gives rise to internal representations which allow for precise locomotor adjustments and thus safe navigation through one’s environment. Our findings constitute an important link between previous studies investigating echo-acoustic obstacle detection and localization under laboratory conditions on the one hand, and the real-life practicality of this information for blind people in evoking precise motor responses for collision avoidance on the other hand.

## 6. Conclusion

In a 2AIFC experiment, we showed that sighted human subjects can be trained to use echo-acoustic cues to discriminate between different orientations in enclosed spaces with high accuracy. For this task, binaural comparison of the perceived echoes was crucial. A second experiment, in which subjects adjusted their orientation in VEAS via rotations, showed that dynamic acoustic cues facilitate echo-acoustic orientation, especially when the subjects are moving relative to the fixed VEAS and not vice versa. Thus, the current study shows that vestibular and proprioceptive information facilitates echo-acoustic orientation in humans.

## Supplementary Material

A video recording of a blind echolocation expert doing an orientation task in virtual echo-acoustic space. PDF containing the raw data from which all analyses were performed and figures drawn

## Data accessibility

The datasets supporting this article have been uploaded as part of the electronic supplementary material.
