# Supplementary material for: Self-motion facilitates echo-acoustic orientation in humans
Source: R Soc Open Sci. 2014 Nov 12;1(3):140185. doi: 10.1098/rsos.140185 (PMC4448837; doi:10.1098/rsos.140185)
Supplement: A video recording of a blind echolocation expert doing an orientation task in virtual echo-acoustic space. PDF containing the raw data from which all analyses were performed and figures drawn [file rsos140185supp1.pdf]

Wallmeier and Wiegrebe: Self-motion facilitates sonar-guided orientation in humans

Electronic supplementary material containing all raw data that is necessary to conduct the analyses and draw the figures presented in the manuscript.

Experiment 1

Raw data in terms of the just noticeable differences in degrees:

**Position L2**	Run1	Run2	Run3
Subject 1	23,0826	26,5846	24,398
Subject 2	28,5296	30,7921	34,992
Subject 3	24,4748	17,4448	19,4954
Subject 4	30,9802	28,0289	25,2954
Subject 5	41,1175	26,6302	32,1309
Subject 6	26,0804	31,359	20,594
Subject 7	18,2744	24,9375	25,7241
Subject 8	28,7789	21,7002	22,1287

**Position M2**	Run 1	Run 2	Run 3
Subject 1	17,7241	11,1291	14,5035
Subject 2	15,246	18,8643	18,2744
Subject 3	9,0591	11,2415	7,6865
Subject 4	7,5382	7,1821	10,0657
Subject 5	9,4322	14,0413	13,2905
Subject 6	8,3188	11,7802	8,8834
Subject 7	9,8462	8,1877	12,1968
Subject 8	11,1677	7,2885	9,1437

**Position L1**	Run 1	Run 2	Run 3
Subject 1	20,196	26,8817	18,3952
Subject 2	19,5728	31,1262	26,1119
Subject 3	22,2805	16,8912	15,4901
Subject 4	23,1891	16,7706	23,958
Subject 5	35,7937	29,4618	28,0827
Subject 6	16,7516	24,9729	18,892
Subject 7	24,6416	32,9143	22,5205
Subject 8	32,2468	24,6013	37,1341

**Position M1**	Run 1	Run 2	Run 3
Subject 1	8,1431	11,4215	10,7169
Subject 2	9,5179	7,1456	11,228
Subject 3	5,0674	6,2453	4,692
Subject 4	5,9782	4,0907	5,1059
Subject 5	9,5736	7,6144	7,15
Subject 6	6,6811	8,12	9,7509
Subject 7	11,8784	9,5736	8,3674
Subject 8	9,0257	11,1291	6,9626

Experiment 2.1

Raw data in terms of the subjects' chosen orientation in degrees:

**Position L2**	Run 1	Run 2	Run 3	Run 4	Run 5	Run 6	Run 7	Run 8	Run 9	Run 10
Subject 1	18,2	21,4	-0,2	28,6	11,6	19,4	9,6	18,4	12,6	19,2
Subject 2	26,2	-4,8	31,6	15,8	13,4	12,6	15,4	25,8	17,4	24,2
Subject 3	17,2	-1,4	13,8	4,4	11,6	-2,6	9,2	6,8	13,6	7,4
Subject 4	8,4	21,2	7,6	-3,6	12,8	14,8	5,4	16,2	11,4	9,6
Subject 5	23,8	19,2	-3,6	7,8	16,8	5,2	24,8	12,4	21,2	13
Subject 6	17	0,6	7	33,8	8,8	14,8	18,8	8,6	16,4	10
Subject 7	-3,6	-20,8	18	25,2	-0,2	7,4	9,2	11,6	16,4	9,4
Subject 8	-13,8	10,6	-5,4	18,2	23	16,6	12,2	9	15,2	11,4

**Position M2**	Run 1	Run 2	Run 3	Run 4	Run 5	Run 6	Run 7	Run 8	Run 9	Run 10
Subject 1	-166,2	-11,4	-12,4	7	164,8	-12,4	-177,2	-9,2	-2,6	-7,2
Subject 2	155,2	172,8	-7,2	179,6	-178,6	17,4	174	12,2	9,4	6,2
Subject 3	3	174,6	-171	2,4	0,6	-14,8	8,6	5,2	4,6	12,2
Subject 4	6,4	177,8	-14,2	172,2	25,2	172	-178	5,4	-3,2	3,6
Subject 5	171,6	0,2	-167,2	178,8	-0,4	-163,8	12,2	-6,4	-8,2	-4,8
Subject 6	177,2	-171,8	14	5,2	-9,6	3,2	6,4	-4,2	-6,8	2,4
Subject 7	175	13,4	168,2	-21	18,2	-169	-9,4	7,2	1,8	5
Subject 8	-174,2	-175	18,2	17,8	-15,4	-6,6	12	-4,8	3	-5,8

**Position L1**	Run 1	Run 2	Run 3	Run 4	Run 5	Run 6	Run 7	Run 8	Run 9	Run 10
Subject 1	35,2	11,6	17,4	-4	31,8	-5,6	10,2	8,8	17,4	14,6
Subject 2	19,8	-2,4	32,6	28,4	17,6	9,2	9,4	22,8	16,2	13,2
Subject 3	8,6	-4,6	17,8	10,2	3,8	-5,4	11,2	7,8	12,4	7,4
Subject 4	10,8	13,2	-4,6	11,4	7,2	22,6	-2,4	12,2	7,8	11,8
Subject 5	24,8	-2,8	31,4	17,4	-4,8	15,8	25,2	14,4	19,2	10,8
Subject 6	-7,8	2,2	10,4	4,4	22	9,6	-1,6	8,6	7,4	13,4
Subject 7	-23	15	-5	-7,8	10,8	22,2	2,2	11,4	18,6	10
Subject 8	24,8	-4,2	13,6	-5,8	8,2	3	15,8	11,8	13,4	20,2

**Position M1**	Run 1	Run 2	Run 3	Run 4	Run 5	Run 6	Run 7	Run 8	Run 9	Run 10
Subject 1	9,4	-7,8	4,6	8,2	-5,8	-10,2	2,4	6,2	8,4	-0,2
Subject 2	11,4	-6,2	8,4	5,6	-4,4	7,8	-5,2	-2,2	-8,2	-3,2
Subject 3	1,6	3,4	-2,2	-8,4	0,8	-4,6	2,6	3,2	-1,4	2,2
Subject 4	2,6	4,8	-7,2	-4,6	2,2	4,2	-6,2	2,4	-4	-1,6
Subject 5	9,8	12	8,2	-1,2	3,6	9,6	-11,2	5,4	2,8	4,2
Subject 6	-0,4	15,6	-6	0,4	-14,2	-5,4	-1,2	4,8	-4,2	-3,2
Subject 7	-2	5,2	-13,4	-9	13	-8,2	4,6	-5,2	2,8	3,4
Subject 8	-11,4	15,4	9,6	-1	-11,8	2,4	-8	-1,2	6,8	4,4

Experiment 2.2

Raw data in terms of the subjects' chosen orientation in degrees:

**Position L2**	Run 1	Run 2	Run 3	Run 4	Run 5	Run 6	Run 7	Run 8	Run 9	Run 10
Subject 1	14,4	-3,6	7,8	13,6	-5	17,6	-8,6	5,2	2,8	9,2
Subject 2	-10,8	15,6	-21,8	9,8	3,2	14,2	14,2	4,8	12,4	5,2
Subject 3	8,6	-1,8	5,6	3,4	6,2	2,6	-4,6	3,2	-2	2,6
Subject 4	-2,6	4,2	-3,4	-8	5,2	6,4	7,2	-1,2	5,4	-2,8
Subject 5	13,2	-9,4	8,2	5,6	7,6	-12,8	9,2	8,4	3,4	11,2
Subject 6	5,4	11,8	-8,8	4	9,2	-2,8	8,2	3,2	-2,6	6,4
Subject 7	4,2	7,4	4,4	6,2	-4,2	9,8	-4,4	5,8	-2,8	-2,2
Subject 8	11,6	9,6	-5,8	-10	15	7,6	5,2	-0,4	3,6	2,4

**Position M2**	Run 1	Run 2	Run 3	Run 4	Run 5	Run 6	Run 7	Run 8	Run 9	Run 10
Subject 1	-13,6	-168,6	-6	5	166	174,2	7,6	-1,2	6,4	8,2
Subject 2	19,8	1,4	-174	-9,6	-178,2	12,6	-13,2	-2,8	-10,2	-1,4
Subject 3	166,6	13,2	5	168,8	11,4	-4,8	5,6	4,2	-1,8	-3,4
Subject 4	7,2	-162,4	-2,4	1,2	15	-175	9,2	3,2	2,4	2,6
Subject 5	-17,3	14,4	167,2	170,2	-12,8	-174	-9,2	10,4	2,8	4,2
Subject 6	-172,6	23,4	-168,2	-2,8	13,2	1,2	-11,4	9,8	5,6	2,8
Subject 7	12,8	-174,8	161,4	-26,2	-8,2	5	11,8	-6,4	2,2	-3,2
Subject 8	169,8	-172,8	-9,6	19,2	3,6	-10,4	15,8	-4,6	3,6	-4,8

**Position L1**	Run 1	Run 2	Run 3	Run 4	Run 5	Run 6	Run 7	Run 8	Run 9	Run 10
Subject 1	13,8	5,4	-4,4	19,2	8,6	13,2	-5,2	7,2	2,4	10,8
Subject 2	5,6	21,4	15,8	-4	7,4	-6,8	15,4	10,2	6,6	3,2
Subject 3	5,2	-3,8	7,8	3,4	-1,4	6,6	7,2	3,6	-2,4	4
Subject 4	-4,4	7,6	2,4	9,6	3,2	3,8	-5,6	-1,2	5,4	2,2
Subject 5	18,6	10,8	-5,6	9,2	6,4	3,4	-7,8	-1,6	6,8	5,2
Subject 6	-8,4	-3	12,2	2,8	13,4	2,4	5,2	-0,6	4,6	-3,2
Subject 7	11	5,6	-6,4	5,8	15,6	5,8	2,6	3,8	-1,4	8,2
Subject 8	-4,2	-2	14,6	6,6	-5,2	-2,2	9,2	-4,6	4,2	3,6

**Position M1**	Run 1	Run 2	Run 3	Run 4	Run 5	Run 6	Run 7	Run 8	Run 9	Run 10
Subject 1	7,6	10,2	-9,6	5,4	-7,8	5,2	-6,4	5	4,4	-3,8
Subject 2	11,4	6,2	-9,4	5,2	7	-8,8	-5,2	4,4	-4,8	-1,4
Subject 3	-3,2	4,6	0,2	-1,4	-2	-5,8	2,2	-3,2	2,4	1,6
Subject 4	5,8	-2,6	-1,2	6,4	3,2	4,2	2,8	-3,6	4,3	2,6
Subject 5	-7,4	-11,4	1,2	-5	9,6	3,6	6,2	2,2	6,4	5,8
Subject 6	-3,6	-6,4	9,8	6,2	-2	3,2	-7	4,8	-4,2	3,6
Subject 7	0,4	7,8	-8,2	5,4	-6,6	6,8	-8,6	3	-4,4	-5,8
Subject 8	-2,6	-0,8	11,6	-3	7,4	4,2	-7,2	-0,4	4,2	3,6

Experiment 2.3

Raw data in terms of the subjects' chosen orientation in degrees:

Position L2	Run 1	Run 2	Run 3	Run 4	Run 5	Run 6	Run 7	Run 8	Run 9	Run 10
Subject 1	7,4	15,8	-4,6	9,4	6,2	4,8	-7,6	8,4	4	6,2
Subject 2	8,2	-3,8	9,6	13,2	11,8	1,4	-1,8	5,8	6,4	3,4
Subject 3	-2,2	4,8	3,4	1,8	-1,2	-3,6	5,8	2,2	-3,4	3,6
Subject 4	5,6	-3,2	-2,6	5,4	3,8	-4,4	6,2	-2,4	-3,4	4,6
Subject 5	13,2	-4	5,6	7,2	-2,4	5,8	-4,2	9,4	6,2	-0,2
Subject 6	-2,2	5,6	13,2	4,4	7,8	2,4	-0,2	3,6	1,8	5,4
Subject 7	10,6	4,2	6,8	-1,2	7	3,2	6,4	-0,6	2,8	3,2
Subject 8	7,6	-1,4	11,2	4,6	2,8	7,2	3	-4,4	-1,2	3,4

Position M2	Run 1	Run 2	Run 3	Run 4	Run 5	Run 6	Run 7	Run 8	Run 9	Run 10
Subject 1	2,2	24,2	-14,4	-2,4	6	1	-10,4	8,2	3,6	5,2
Subject 2	11,4	-2,2	-12,2	-179	4,6	-13,4	15,2	-7,2	-3,2	-5,6
Subject 3	9,6	-5,6	3	-14	-2,8	4,6	-3,6	2,2	3,8	-2,6
Subject 4	-7,2	-10,2	-0,8	7	9,6	-14,2	-6,8	3,4	-5,2	-4,6
Subject 5	2,8	-9,6	-168,2	-23,2	-5,4	6,6	12,8	-7,2	2,2	-4,6
Subject 6	-7,6	4,2	-9,6	13,4	5,2	9,2	-4,8	3,4	1,6	6,2
Subject 7	3,2	168,4	11,8	-4,8	6,6	4,2	7,8	-3,2	1,8	-3,6
Subject 8	-13,6	11	-2,8	9,2	7,4	-4,6	0,8	-2,4	-4,6	3,2

Position L1	Run 1	Run 2	Run 3	Run 4	Run 5	Run 6	Run 7	Run 8	Run 9	Run 10
Subject 1	12,2	13,8	-3,2	4,6	8,4	-2,4	7	6,2	7,4	-0,8
Subject 2	11,6	9,2	1,6	8,8	7,2	5,8	-2,2	11,2	2,6	3,2
Subject 3	3,6	4,4	-5,8	0,4	2,2	3,4	-4,2	2,6	3,2	-2,2
Subject 4	2,4	6,8	5,4	-4,6	3,8	3,2	5,8	3,4	-1,2	2,6
Subject 5	12,4	-2,6	7,4	10,4	5	7,6	2,8	6,6	-1,8	5,8
Subject 6	8,6	17,2	7,2	5,4	-3	7,8	4,4	5,2	-0,4	6,2
Subject 7	10	-1,4	3,2	11,8	5,6	4,4	2,8	7,4	2,2	-1,8
Subject 8	5,2	11,6	-2,2	5,4	12,6	-3	9,6	-3,4	-2,8	4

Position M1	Run 1	Run 2	Run 3	Run 4	Run 5	Run 6	Run 7	Run 8	Run 9	Run 10
Subject 1	6,4	11,2	-7,4	-12,4	8,2	5,6	2,2	-4,8	2,4	-5,2
Subject 2	-8,6	2,4	4,4	-3	10,2	-5,8	-4,2	5,4	6,8	-2,4
Subject 3	0,8	-2,6	-3,2	4,6	1,8	2,4	-5,6	2,2	1,4	-3,4
Subject 4	8,2	-4,6	3,8	4,2	-2,2	6,2	-1,2	-3,6	-2,4	1,6
Subject 5	7,6	-6,4	-10,2	-5,4	4,2	-3,8	5	6,4	-2,2	5,2
Subject 6	-6,2	3,4	1,4	-8,2	3,6	5,2	-4,4	2,6	-1,4	3,2
Subject 7	3,8	-6,2	-9	-5,8	3,4	-2,6	4,8	2,2	4	1,2
Subject 8	-14,4	8,2	6,4	4,8	-1,6	3,2	9,4	-4,2	-1,8	-2,2
